# Sex Differences in Risk Factors for Cardiovascular Disease: The PERU MIGRANT Study

**DOI:** 10.1371/journal.pone.0035127

**Published:** 2012-04-05

**Authors:** Antonio Bernabe-Ortiz, Catherine Pastorius Benziger, Robert H. Gilman, Liam Smeeth, J. Jaime Miranda

**Affiliations:** 1 CRONICAS Center of Excellence in Chronic Diseases, Universidad Peruana Cayetano Heredia, Lima, Peru; 2 Epidemiology Unit, School of Public Health and Administration, Universidad Peruana Cayetano Heredia, Lima, Peru; 3 University of Minnesota Medical School, Minneapolis, Minnesota, United States of America; 4 Department of International Health, Johns Hopkins Bloomberg School of Public Health, Baltimore, Maryland, United States of America; 5 Área de Investigación y Desarrollo, A.B. PRISMA, Lima, Peru; 6 Faculty of Epidemiology and Population Health, London School of Hygiene and Tropical Medicine, London, United Kingdom; 7 Department of Medicine, School of Medicine, Universidad Peruana Cayetano Heredia, Lima, Peru; College of Pharmacy, University of Florida, United States of America

## Abstract

**Introduction:**

Although men and women have similar risk factors for cardiovascular disease, many social behaviors in developing countries differ by sex. Rural-to-urban migrants have different cardiovascular risk profiles than rural or urban dwellers. The objective of this study was to evaluate the sex differences with specific cardiovascular risk factors in rural-to-urban migrants.

**Methods and Results:**

We used the rural-to-urban migrant group of the PERU MIGRANT cross-sectional study to investigate the sex differences in specific cardiovascular risk factors: obesity, hypertension, metabolic syndrome, as well as exposures of socioeconomic status, acculturation surrogates and behavioral characteristics. Logistic regression analysis was used to characterize strength of association between sex and our outcomes adjusting for potential confounders. The sample of migrants was 589 (mean age 46.5 years) and 52.4% were female. In the adjusted models, women were more likely to be obese (OR=5.97; 95%CI: 3.21–11) and have metabolic syndrome (OR=2.22; 95%CI: 1.39–3.55) than men, explaining the greatest variability for obesity and metabolic syndrome but not for hypertension.

**Conclusions:**

Our results suggest that interventions for CVD in Peru should be sex-specific and address the unique health needs of migrant populations living in urban shantytowns since the risk factors for obesity and metabolic syndrome differ between males and females.

## Introduction

Cardiovascular diseases are rapidly becoming the leading causes of morbidity and mortality in low- and middle-income countries (LMIC) [1]. In these settings, urbanization, due mainly to internal migration, is one of the main contributors to the transition towards increasing CVD [2], [3], [4]. Thus, rise in cardiovascular diseases in LMIC can be attributed to changes in social and health behaviors, such as physical inactivity, decreased fruit intake and increased intake of energy dense foods and salt, as well as tobacco use and alcohol consumption [5], [6].

Rural-to-urban migrants have different cardiovascular risk profiles than rural or urban dwellers; yet, most of these studies are limited to international migrant pattern from developing to developed countries, particularly those residing in the United States. Few studies have addressed the impact of rural-to-urban migration on cardiovascular outcomes in LMIC, e.g. the Kenyan Luo migration study [7], Yi People Study in China [8], Indian Migration Study in India [9], and the PERU-MIGRANT study in Peru [10]. Recently, a systematic review of rural-to-urban migration studies concluded that several cardiovascular risk factors are higher or more common in migrants than in rural groups, but lower or less common than in urban groups [11].

While men and women have similar risk factors for cardiovascular diseases, many social behaviors in LMIC differ by sex. For example, women who smoke have three times the risk of heart attacks and have their first heart attacks much earlier than men and woman non-smokers [12]. A Brazilian study demonstrated that high cholesterol and hypertension were more prevalent among women compared to men [13]. In Peru, three studies have shown that metabolic syndrome, abdominal obesity, and low high density lipoprotein-cholesterol (HDL) are higher in women than men while there seems to be no differences in hypertension, hypertriglyceridemia or high fasting glucose [14], [15], [16].

Reliable estimates of prevalence, patterns and population distribution of cardiovascular risk factors in Latin American populations are needed in order to design effective treatment programs. The PERU MIGRANT study found that the impact of rural-to-urban migration on cardiovascular risk profile was not uniform across different risk factors, and was further influenced by the age at which migration occurred [10]. Moreover, a gradient was observed for some risk factors across study groups. The objectives of this study were to evaluate the sex differences in exposures to socioeconomic, acculturation and behavioral characteristics and assess the impact of sex on specific cardiovascular risk factors (obesity, hypertension, and metabolic syndrome) in a rural-to-urban migrant population.

## Methods

### Study Design, Setting, and Participants

The general objectives and design of the PERU-MIGRANT study have been previously published [17]. Briefly, a cross-sectional survey was performed using a single-stage random sampling and involving migrants from rural to urban areas in Peru. Potential participants, born in the rural setting of Ayacucho, who migrated to, and were currently living in the urban setting of Lima, were asked to participate. Ayacucho is a region of Peru located in the south-central Andes. Its capital is the city of Ayacucho and is relatively closed to Lima (8 hours by bus). The region was one of the hardest hit by terrorism, whose population massively migrated to coastal cities, especially to Lima.

The area called “Las Pampas de San Juan de Miraflores" was the setting where this study was undertaken. The sampling frame for this migrant group was the local census performed in 2000, updated in 2006, to identify all those who stated they had been born in the Department of Ayacucho and were currently living in Lima. The sample was stratified by sex and age groups to reduce the confounding due to these variables.

### Data Collection

A team of community health workers was trained to enroll participants and to conduct the questionnaires assessing socio-demographic, acculturation and behavioral variables. As part of this evaluation, participants were invited to a clinic visit where height, weight, waist circumference (WC), systolic (SBP) and diastolic (DBP) blood pressure, as well as blood samples were obtained using standardized methods and calibrated tools [17]. Total cholesterol, triglycerides and HDL were measured in serum, whereas fasting glucose was assessed in plasma.

### Outcomes and Variable Definitions

Three were our outcomes of interest: obesity, hypertension, and metabolic syndrome. Obesity was defined as body mass index (BMI) ≥30 kg/m^2^ for men and women according to accepted guidelines [18]. Hypertension was considered whether SBP ≥140 and/or DBP ≥90 mm Hg, or self-report of physician diagnosis and currently receiving anti-hypertensive medication [19], [20]. Because of established ethnic differences, we followed the International Diabetes Federation’s (IDF) cut-off points specific for non-European populations [21]. Thus, abdominal obesity according to IDF for our South American population was WC ≥90 cm (men) or ≥80 cm (women) [21], [22]. In addition to this, we also followed the Latin American Consortium in Obesity Studies (LASO) cut-off criteria of WC ≥97 cm (men) or ≥94 cm (women) [23], [24]. Finally, according to the harmonized criteria participants having 3 or more of the following criteria were defined as having metabolic syndrome: (1) abdominal obesity (ethnic specific cut-offs of WC >90 cm in men and >80 cm in women); (2) triglycerides ≥150 mg/dL or drug therapy for elevated triglycerides; (3) low HDL-cholesterol (<40 mg/dL in men and <50 mg/dL in women) or drug therapy for low HDL cholesterol; (4) SBP ≥130 mm Hg, DBP ≥85 mm Hg, or current pharmacologic treatment for hypertension; (5) fasting glucose ≥100 mg/dL or current antidiabetic medication use (insulin or oral agents) [21]. A detailed description of the socio-demographic, acculturation and behavioral variables is shown in File S1.

### Statistical Analysis

STATA 11 for Windows (STATA CORP, College Station, Texas, USA) was used for all analyses. Initially, a brief description of the socio-demographic, acculturation, behavioral, and clinical variables was performed according to sex. Frequencies and percentages were used to present categorical variables. Then, age-adjusted odds ratios (OR) and 95% confidence intervals (95%CI) were estimated using logistic regression (crude model). Finally, the strength of the association between sexes (male as reference) and our outcomes of interest were estimated adjusting for potential confounders. Diverse models were generated including different confounders such as socio-demographic variables (education level, socioeconomic status using household assets); acculturation surrogates (language spoken at home, language preferences for listening, age at first migration, and lifetime urban exposure); and behavioral variables (smoking, alcohol consumption, and physical activity level).

### Ethics

Ethical approval for this protocol was obtained from Institutional Review Boards at Universidad Peruana Cayetano Heredia in Peru and London School of Hygiene and Tropical Medicine in the UK. All enrolled participants gave written informed consent.

## Results

A total of 872 potential participants were contacted an asked to participated, but only 589 (participation rate: 67.5%) completed the survey. The sample of migrants was 589; 52.5% were females, on average the sample age was 47.8 years (SD±11.7), and had a mean of age of first migration of 14.7 years (inter-quartile range, IQR: 10–17).

### Sex Differences in Cardiovascular Risk Profile

Demographic, socioeconomic status (SES), acculturation, and behavioral variables stratified by sex are shown in Table 1. Compared to women, men were more likely to have higher socioeconomic status –completed secondary or higher education and household assets–, to use Spanish as the language of preference at home and more likely to be current smokers and binge drinkers. Women were more likely to be moderately physically active, whereas men were more likely to reported heavy physical activity.

**Figure 1:**
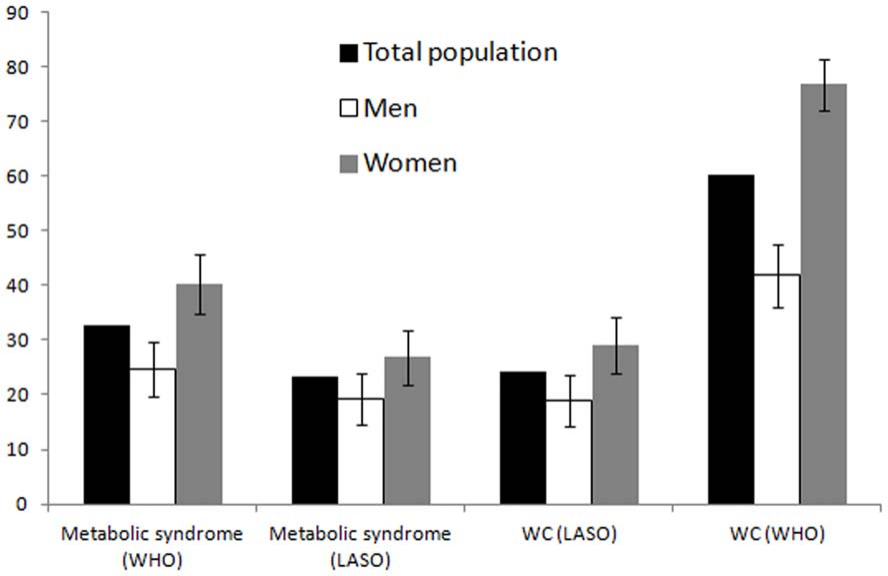
Prevalence of Metabolic Syndrome by different waist circumference cutoffs by sex (n=589). *Based on IDF WC cut-off criteria [WC ≥90 cm (men) or ≥80 cm (women)]. **Based on LASO WC cut-off criteria [WC ≥97 cm (men) or ≥94 cm (women)].

Regarding our outcomes of interest, women had greater prevalences of obesity, assessed by BMI, and metabolic syndrome, whereas hypertension prevalence was similar. Women were more likely to be obese by BMI and WC definitions (Figure 1). According to metabolic syndrome components, abdominal obesity and low HDL were more common among females, whereas high blood pressure was more frequent among males (Table 2).

**Table 1:** Socio-economic, acculturation and behavioral characteristics of the study population by sex.

Characteristics			Total	Male	Female	p-value*
			(n=589)	(n=280)	(n=309)	
Age groups	30–39		164 (27.8)	67 (23.9)	97 (31.4)	0.23
	40–49		173 (29.4)	89 (31.8)	84 (27.2)	
	50–59		167 (28.4)	82 (29.3)	85 (27.5)	
	60+		85 (14.4)	42 (15.0)	43 (13.9)	
**Socioeconomic Status variables:**						
Education	None/some primary		183 (31.1)	46 (16.5)	137 (44.3)	<0.001
	Primary completed		99 (16.8)	51 (18.3)	48 (15.5)	
	Secondary or higher		306 (52.0)	182 (65.2)	124 (40.1)	
Household Assets	Lowest tertile		119 (20.2)	47 (16.8)	72 (23.3)	0.002
	Middle		253 (43.0)	110 (39.3)	143 (46.3)	
	Highest tertile		217 (36.8)	123 (43.9)	94 (30.4)	
**Acculturation variables:**						
Language spoken at home		Spanish and Quechua	48 (8.2)	15 (5.4)	33 (10.8)	0.02
		Spanish only	536 (91.8)	263 (94.6)	273 (89.2)	
Language preference	Spanish and Quechua		407 (70.4)	194 (70.0)	213 (70.8)	0.85
	Spanish only		171 (29.6)	83 (30.0)	88 (29.2)	
Age at first migration	≤12 years		225 (38.5)	106 (38.3)	119 (38.6)	0.93
	>12 years		360 (61.5)	171 (61.7)	189 (61.4)	
Urban exposure	Lowest tertile		188 (33.6)	86 (32.5)	102 (34.7)	0.77
	Middle		186 (33.3)	92 (34.7)	94 (32.0)	
	Highest tertile		185 (33.1)	87 (32.8)	98 (33.3)	
**Behavioral variables:**						
Smoking	Nonsmoker/former smoker		530 (90.0)	229 (81.8)	301 (97.4)	<0.001
	Current smoker		59 (10.0)	51 (18.2)	8 (2.6)	
Alcohol consumption	None		129 (21.9)	40 (14.3)	89 (28.8)	<0.001
	Regular drinker		412 (70.0)	197 (70.4)	215 (69.6)	
	Binge drinker		48 (8.1)	43 (15.4)	5 (1.6)	
Physical activity	Low		173 (20.7)	76 (27.4)	97 (31.8)	<0.001
	Moderate		211 (36.3)	78 (28.2)	133 (43.6)	
	Heavy		198 (34.0)	123 (44.4)	75 (24.6)	

* Chi squared test was used to compare characteristics by sex.

**Table 2:** Clinical characteristics of the study population by sex.

Characteristics	Total	Male	Female		p-value*
	N=589	N=280	N=309		
**Obesity**					
Percentage (95%CI)	21.1 (17.8–24.4)	10.7 (7.1–14.4)	30.4 (25.3–35.6)		<0.001
**Hypertension**					
Percentage (95%CI)	13.1 (10.4–15.8)	13.6 (9.6–17.7)	12.6 (8.9–15.8)		0.72
**Metabolic Syndrome components**					
**Abdominal obesity**					
Percentage (95%CI)	60.1 (56.1–64.1)	41.8 (36.0–47.6)	76.7 (72.0–81.4)		<0.001
**High triglycerides**					
Percentage (95%CI)	41.6 (37.6–45.6)	41.1 (35.3–46.9)	42.1 (36.5–47.6)		0.81
**Low HDL-cholesterol**					
Percentage (95%CI)	57.9 (53.9–61.9)	43.2 (37.4–49.1)	71.2 (66.1–76.3)		<0.001
**High blood pressure**					
Percentage (95%CI)	22.6 (19.2–26.0)	26.8 (21.6–32.0)	18.8 (14.4–23.1)		0.02
**High fasting glucose**					
Percentage (95%CI)	8.7 (6.4–10.9)	7.1 (4.1–10.2)	10.0 (6.7–13.4)		0.21
**Total Metabolic Syndrome**					
Percentage (95%CI)	32.8 (29.0–36.6)	24.6 (19.6–29.7)	40.1 (34.6–45.6)		<0.001

* Chi squared test was used to compare characteristics by sex.

HDL; high-density lipoprotein.

### Determinants of Cardiovascular Risk Factors

In the multivariable models, after adjusting for other potential factors, sex was an independent predictor for obesity and metabolic syndrome (Table 3) and women were almost six times more likely to be obese and twice as likely of having metabolic syndrome. There was no evidence of an association between sex and hypertension; however, further adjustment for acculturation variables and also alcohol consumption provided strong evidence of an association between sex and hypertension. Nevertheless, this association was attenuated in the fully adjusted model (Table 3).

**Table 3:** Relationship of cardiovascular disease risk factors with socioeconomic, acculturation, behavioral variables with male as reference (n=567).

	Variables	Obesity	Hypertension	Metabolic Syndrome
		OR (95%CI)	OR (95%CI)	OR (95%CI)
Model 1	Sex†	**3.76 (2.37–5.97)**	1.55 (0.98–2.44)	**2.31 (1.59–3.36)**
**Model 1 plus Socioeconomic status (SES) variables**				
Model 2	Education	**3.28 (2.01–5.36)**	1.42 (0.87–2.33)	**2.15 (1.43–3.22)**
Model 3	Assets	**3.73 (2.34–5.94)**	1.47 (0.92–2.33)	**2.33 (1.59–3.40)**
Model 4	**All SES variables**	**3.25 (1.99–5.31)**	1.38 (0.84–2.26)	**2.16 (1.44–3.24)**
**Model 1 plus Acculturation variables**				
Model 5	Language at home	**3.85 (2.37–6.24)**	**1.69 (1.04–2.74)**	**2.23 (1.51–3.29)**
Model 6	Language preference for listening	**3.87 (2.39–6.28)**	**1.71 (1.06–2.76)**	**2.26 (1.53–3.34)**
Model 7	Age at migration	**3.95 (2.43–6.40)**	**1.73 (1.07–2.80)**	**2.30 (1.56–3.39)**
Model 8	Lifetime urban exposure	**4.11 (2.52–6.71)**	**1.72 (1.07–2.78)**	**2.34 (1.58–3.45)**
Model 9	**All acculturation surrogates**	**4.07 (2.49–6.66)**	**1.69 (1.04–2.75)**	**2.32 (1.56–3.45)**
**Model 1 plus Behavioral variables**				
Model 10	Smoking	**4.13 (2.52–6.77)**	1.55 (0.96–2.50)	**2.17 (1.47–3.19)**
Model 11	Alcohol consumption	**4.87 (2.94–8.08)**	**1.72 (1.05–2.82)**	**2.25 (1.52–3.34)**
Model 12	Physical activity	**4.02 (2.50–6.48)**	1.47 (0.91–2.35)	**2.37 (1.61–3.49)**
Model 13	**All behavioral variables**	**5.50 (3.20–9.46)**	1.61 (0.95–2.70)	**2.19 (1.44–3.31)**
**Final models**				
Model 14	**Model 4 + Model 9 + Model 13**	**5.97 (3.21–11.11)**	1.63 (0.90–2.94)	**2.22 (1.39–3.55)**

All odds ratios are adjusted for age.

Bold=p<0.05.

† Male sex is the reference.

## Discussion

In this study, using rural-to-urban migrant data, we observed important differences according to sex in the prevalence of obesity, metabolic syndrome, and hypertension after adjusting for well-known risk factors. The present analysis expands on previous observations by taking advantage of proxies for acculturation in the setting of rural-to-urban within-country migration and its relationship with major cardiovascular risk factors. Thus, our results are consistent with the literature, including other Peruvian studies, which have shown that metabolic syndrome and obesity are higher in women than men while there seems to be no differences in hypertension [14], [15], [25]. However, a previous report involving Brazilian population found that hypertension was more prevalent among women compared to men [13].

Regarding metabolic syndrome components, prevalence differences in this population are mainly due to abdominal obesity and low HDL among women than elevated blood pressure, which was more common among men. These results are compatible to previous studies [16], [26], [27]. In addition, a preliminary work in a similar socioeconomic setting of urban poverty showed that women are less likely to seek help for symptoms compatible to acute coronary syndrome [28]. Furthermore, our results suggest that women might be in disadvantage facing cardiovascular disease.

The reported prevalence of obesity in Latin American populations varies greatly (9.9% to 35.7%) [29]. General adiposity and abdominal obesity are associated with an increased risk of death from cardiovascular disease [30]; however, cut-offs derived from other populations for use in our population have been questioned. In the LASO study, waist-to-hip ratio was found to be the most accurate anthropometric indicator for high risk of cardiovascular disease [24]. Obesity has been associated with older age at first migration, language speaking proficiency, and language preferences in migrant population [31]. Thus, these variables were included in our models. After adjusting for acculturation variables, we found female migrants were more likely to be obese, have hypertension and have metabolic syndrome than men. These findings suggest that urbanization and the migration process do indeed have an important influential effect on cardiovascular disease profile, equal or more than socioeconomic factors per se, which differs by sex. Traditionally, migrants tend to be a highly self-selected group, whereby the healthier, wealthier residents of an area migrate to urban areas for better opportunities [32]. In this case, rural-to-urban migrants from Ayacucho (Peru) fled the area due to strong political violence [33] rather than only a migration for economic reasons: the migrants were not simply a small self-selected atypical group.

Of note, among biological markers for metabolic syndrome, a great proportion of women had HDL under the recommended cut-offs [22]. Although, on average, HDL mean was significantly lower among men (data not shown), the proportion of women with low HDL was 71.2%. These findings have been previously reported in different Peruvian women [14], [15], [16] and are also observed in our migrant population.

The study benefits from the use of a well-defined rural-to-urban within-country migrant population, as well as objective measures of anthropometry, biochemical and metabolic markers. However, the study is of limited generalizability because it was conducted in a specific group of migrants from a socially deprived urban part of Lima, Peru. The group of migrants was well established in the area, many having lived there for over 20 years. Due to the cross-sectional nature of the study, we are unable to comment about the causal relationship between sex and migration, and whether the differences are due to migration per se, or the urban environment.

Previous work by our group with the same dataset, examined the differences between the migrant group, and urban and rural dwellers but sex differences were not explored [10]. In addition, the PERU MIGRANT study did not address dietary patterns, contraceptives and hormone therapy use, or menopause status in this population. After menopause, the lipid profile changes in women, with increasing levels of LDL and decreasing levels of HDL cholesterol [34]. The role of sex hormones and how they affect CVD risk factors in this population was not assessed. Finally, our study may have been underpowered to detect some of the sex differences, for example between smoking and hypertension. However, our study involved a great number of migrants and is the first to show associations between sex and cardiovascular risk factors.

### Conclusions

The findings in this rural-to-urban migrant population suggest that sex is an important factor associated with established cardiovascular risk factors. Regarding metabolic syndrome components, abdominal obesity and low HDL-cholesterol are more common among women, whereas elevated blood pressure component is more frequent among men. Secondarily, smoking and alcohol use are similarly more prevalent in men. Thus, treatment and prevention programs should address sex differences, as well as the unique health needs of migrant populations living in urban shantytowns.

## Supporting Information

File S1 Definitions of socio-demographic and behavioral variables.
(DOCX)
