# Supplementary material for: Sex Differences in Risk Factors for Cardiovascular Disease: The PERU MIGRANT Study
Source: PLoS One. 2012 Apr 5;7(4):e35127. doi: 10.1371/journal.pone.0035127 (PMC3320626; doi:10.1371/journal.pone.0035127)
Supplement: File S1 — Definitions of socio-demographic and behavioral variables. (DOCX) [file pone.0035127.s001.docx]

## SUPPORTING INFORMATION S1

## Definitions of socio-demographic and behavioral variables

**Socioeconomic status**

**Household assets:** Weighted-index constructed based on current ownership of household assets (gas cooker, radio, color television, refrigerator, computer, telephone, mobile phone, cable, internet, bicycle, motorcycle, and car) divided into tertiles.

**Acculturation**

**Language preferences:** A scale based on language spoken in the home and language preference on the radio, previously described and validated [1,2,3], was used.

**Age at first migration:** Self-ascertainment of age (years) when first migrated for a period of 6 months or more. The dichotomous variable was created at < or = 12 years or > 12 years as to evaluate the effects of migration before and after puberty.

**Lifetime exposure to urban area:** Number of years lived in an urban area divided over age and split into tertiles.

**Behavioral variables**

**Smoking:** Smoking status current (>100 cigarettes in lifetime and last cigarette <6 months) and nonsmoker [which includes former smokers (>100 cigarettes in lifetime and last cigarette >6 months) and never] by default as they did not meet the requirements for current smoker, defined from an adapted version of the WHO STEPS questionnaires [4].

**Alcohol consumption:** Frequency of alcohol consumption in the last year, volume of alcohol consumption, and frequency of hangover in the last month was defined by an adapted version of the WHO STEPS questionnaires. Abstinent alcohol consumption was defined as never consuming any volume of alcohol and binge drinking was defined as 2 or more nights in the past month and having ever drunk 6 or more drinks at a time.

**Physical activity:** In accordance with the International Physical Activity Questionnaire (IPAQ) protocol, the categorical physical activity levels were coded based on both total days of physical activity and metabolic equivalents (MET) minutes/week. Moderate physical activity was coded as 5 or more days of any combination of walking, moderate-intensity or vigorous-intensity activities achieving at least 600 MET minutes per week. High physical activity was coded as 7 or more days of any combination of walking, moderate-intensity or vigorous-intensity activities achieving a minimum total physical activity of at least 3000 MET minutes/week. Those with low physical activity did not meet the moderate or high physical activity criteria.

**Footnote:**

1. Bernabe-Ortiz A, Gilman RH, Smeeth L, Miranda JJ (2010) Migration surrogates and their association with obesity among within-country migrants. Obesity (Silver Spring) 18: 2199-2203.

2. De Silva MJ, Harpham T, Tuan T, Bartolini R, Penny ME, et al. (2006) Psychometric and cognitive validation of a social capital measurement tool in Peru and Vietnam. Soc Sci Med 62: 941-953.

3. Deyo RA, Diehl AK, Hazuda H, Stern MP (1985) A simple language-based acculturation scale for Mexican Americans: validation and application to health care research. Am J Public Health 75: 51-55.

4. World Health Organization (2008) WHO STEPwise approach to Surveillance (STEPS). Geneva, Switzerland: WHO.
